# U. S. V. M. A.

**Published:** 1898-04

**Authors:** 


### U. S. V. M. A.

President Salmon has appointed the following as a Committee on Charter: Drs. W. Horace Hoskins, T. Bent. Cotton, and A. W. Clement.

Among those who are expected to discuss the various aspects of the meat-inspection question are Dr. Leonard Pearson, as it applies to "the Concentration of Municipal Abattoirs into Large Establishments under Municipal Control;" Dr. W. Horace Hoskins, as to the "Education of the People of Cities and Towns as to the Necessity for Municipal Meat-inspection;" Dr. R. W. Hickman, of New York City, as to "Slaughter-house Inspection;" Dr. C. W. Heitzman, of New Orleans, as to "Retail Market Inspection."

Dr. Ridge's appeal for greater interest among Pennsylvania veterinarians has been sent to five hundred members of the profession in the Keystone State, and this State will send a number of applicants for membership.

Chairman Peters and his committee are already planning for the reception of those who attend the meeting at Omaha. The ladies on the first day will be invited to attend the opening address of the President, and then in chartered cars be transported to the Exhibition Grounds under the guidance of the Nebraska ladies.

A reception to all members and visitors in the evening, with music and light refreshments by the Nebraska Association. Officers of the U. S. and State Associations will form the reception committee.

An excellent feature already under consideration is a noonday luncheon for all in attendance. This better regulates the sessions and keeps the members closer together.

A trip to Council Bluffs and a visit to the large stores and public buildings will be a second day feature for the ladies.

The annual banquet on the second evening, a theatre party or some suitable entertainment for the ladies, is under consideration.

